# Device for Negative Pressure Wound Therapy in Low-Resource Regions: Open-Source Description and Bench Test Evaluation

**DOI:** 10.3390/jcm11185417

**Published:** 2022-09-15

**Authors:** Ramon Farré, Miguel A. Rodríguez-Lázaro, Julian Gonzalez-Martin, Pedro Castro, Teresa Hospital, Yaroslau Compta, Gorka Solana, David Gozal, Jorge Otero

**Affiliations:** 1 Unitat de Biofísica i Bioenginyeria, Facultat de Medicina i Ciències de la Salut, Universitat de Barcelona, 08036 Barcelona, Spain; 2 CIBER de Enfermedades Respiratorias, 28029 Madrid, Spain; 3 Institut Investigacions Biomèdiques August Pi Sunyer, 08036 Barcelona, Spain; 4 Microbiology Department-CDB, Hospital Clinic-ISGlobal-University of Barcelona, 08036 Barcelona, Spain; 5 CIBER of Infectiuos Diseases (CIBERINFEC), Instituto de Salud Carlos III, 28029 Madrid, Spain; 6 Intensive Care Unit, Internal Medicine Department, Hospital Clínic, 08036 Barcelona, Spain; 7 Institut de Neurociències, Service of Neurology, Parkinson’s Disease and Movement Disorders Unit, Hospital Clinic de Barcelona, 08036 Barcelona, Spain; 8 Institut de Neurociències, Maeztu Center, Universitat de Barcelona, 08036 Barcelona, Spain; 9 Faculdade de Engenharias e Tecnologias, Universidade Save, Maxixe, Mozambique; 10 Department of Child Health, The University of Missouri School of Medicine, Columbia, MO 65212, USA

**Keywords:** vacuum pressure therapy, wound healing, low-cost medical device, low-and middle-income countries, open-source hardware

## Abstract

Background: Negative (vacuum) pressure therapy promotes wound healing. However, commercially available devices are unaffordable to most potential users in low- and middle-income countries (LMICs), limiting access to many patients who could benefit from this treatment. This study aimed to design and test a cheap and easy-to-build negative pressure device and provide its detailed open-source description, thereby enabling free replication. Methods: the negative pressure device was built using off-the-shelf materials available via e-commerce and was based on a small pump, a pressure transducer, and the simplest Arduino controller with a digital display (total retail cost ≤ 75 US$). The device allows the user to set any therapeutic range of intermittent negative pressure and has two independent safety mechanisms. The performance of the low-cost device was carefully tested on the bench using a phantom wound, producing a realistic exudate flow rate. Results: the device generates the pressure patterns set by the user (25–175 mmHg of vacuum pressure, 0–60 min periods) and can drain exudate flows within the clinical range (up to 1 L/h). Conclusions: a novel, low-cost, easy-to-build negative pressure device for wound healing displays excellent technical performance. The open-source hardware description provided here, which allows for free replication and use in LMICs, will facilitate the application and wider utilization of this therapy to patients.

## 1. Introduction

The application of negative pressure-based therapy protocols may contribute to enhancing wound healing in many patients. This treatment, which consists in applying subatmospheric pressure (≈50–150 mmHg) to a foam placed inside the wound sealed with an airtight adhesive drape, has been established as a clinical routine since the mid to late 1990s [1,2,3,4,5]. Different mechanisms have been implicated to explain the beneficial effects and therapeutic action of negative pressure in wound healing to include the reduction in the wound area by edge retraction, the stimulation of granulation tissue formation, mechanical wound cleansing by removal of small tissue debris by suction, the reduction in the fluid concentration of wound healing-impairing proteases, the need for fewer dressing changes because the exudate is drained, and improvement of microcirculation, stimulation of blood flow and oxygenation associated with interstitial edema reduction [1,6,7]. Interestingly, several studies provide evidence that intermittent cycling, rather than continuous vacuum pressure may be advantageous for wound healing regarding blood flow, angiogenesis, tissue oxygenation, and pain reduction [8,9,10].

Negative pressure devices for wound healing are widely available and are manufactured and commercialized by different medical equipment companies. Similar to other types of medical devices, the products offered by the market are mostly built and distributed by international companies and their cost has been tailored and is therefore well-suited and competitive for users in developed countries (https://www.gminsights.com/industry-analysis/negative-pressure-wound-therapy-market; accessed on 15 September 2022). Nevertheless, these devices are not affordable for most hospitals in low- and middle-income countries (LMICs) for either daily clinical routine or even for exceptional circumstances such as war [11,12] or natural disasters [13,14,15]. Different proposals have been published for the implementation of a low-cost application solution involving constant negative pressure for wound healing. In some instances, the negative pressure is obtained from the hospital wall-vacuum system [16,17]. Other authors have proposed the employment of a bellows hand pump [14] or a common aquarium pump as a negative pressure source [15,18]. Clinical tests have shown that such simplified alternatives are useful [16,17,18], and their use when necessary has been encouraged [19]. However, the low-cost options proposed to date have two major limitations: they do not allow for the application of automatically controlled patterns of intermittent negative pressures, and the technical description provided by the authors is not sufficiently detailed for straightforward replication by other users.

Accordingly, this study aimed to design and assess a high-performance, low-cost intermittent negative pressure device for wound therapy, while providing open access to detailed technical information, thereby allowing for public free unrestricted replication. To ascertain the adequate performance of the device, a bench test was carried out using a realistic exudate-secreting wound phantom.

## 2. Materials and Methods

**Figure 1:**
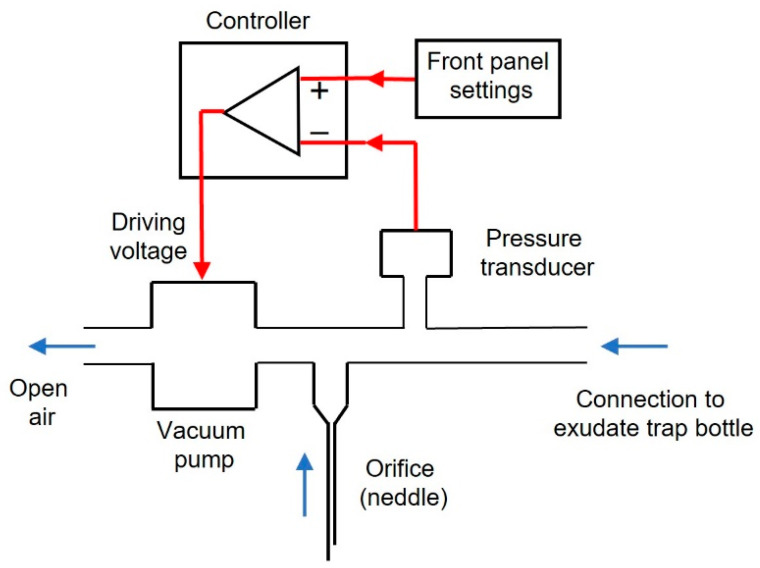
Diagram of the negative pressure device. Black: hardware components, red lines: signals, to generate negative pressure. A transducer measures actual pressure. The user sets the blue arrows: air flow direction. With the outlet open to room air, the vacuum pump suctions air from the wound dressing target pressure in the front panel. The controller compares the target and real pressure and drives the pump with the voltage required to minimize pressures difference. An orifice open to the atmosphere (conventional hypodermic needle) ensures that even if the pump is blocked, the wound will not remain under permanent negative pressure. See text for details.

The devised low-cost negative pressure device for wound healing was assembled with off-the-shelf components purchased via e-commerce sources (Amazon.com, Ebay.com, and Alibaba.com). The setting (Figure 1) is based on a negative pressure pump (Dewin, model JYA-03154, DC-12 V, 60 g, 2~3.2 L/min, vacuum pressure ≤420 mmHg; Xi’an, China). To generate the target vacuum pressure selected by the user, the voltage supplied to the pump was determined by an Arduino Nano microcontroller driven by the real-time signal measured by a negative pressure transducer (−30–0 kPa; XGZP6847030KPGN, CFSensor, Wuhu, China). All technical details of the device, with circuits description, layouts, connections diagram, components list, driving Arduino code, and optional enclosure to be made by any conventional 3D printer, are available for release under free terms following the open-source hardware approach in the Supplementary material.

**Figure 2:**
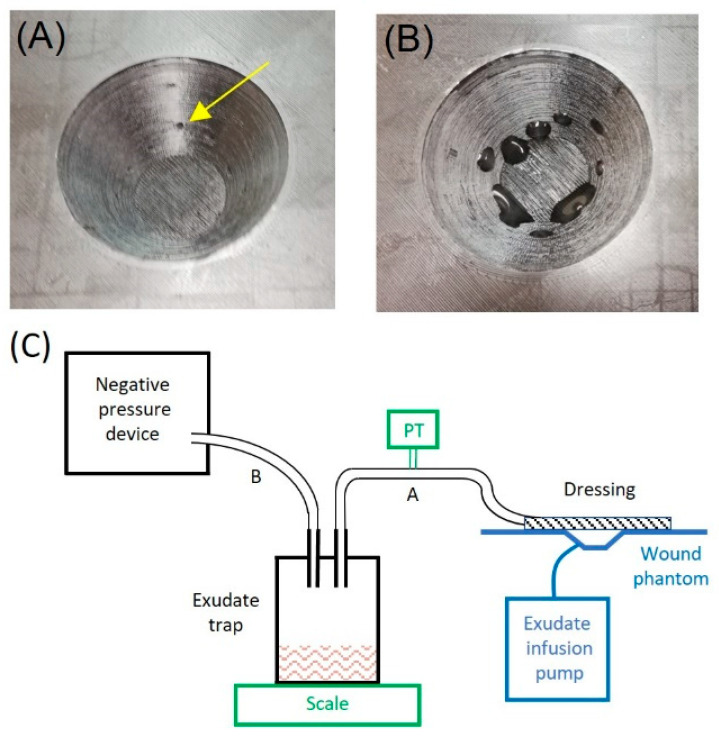
Detailed picture of the 3D printed wound phantom: (**A**) shows the orifices for exudate secretion (one of them noted by a yellow arrow); (**B**) shows exudate secretion by means of an infusion pump. (**C**) Bench test diagram. The functional setting (in black) consists of the negative pressure device connected to the wound phantom (in blue) through a liquid bottle trap for the exudate by flexible tubes A and B and a conventional wound dressing. Two added measuring devices (in green) were added only for bench testing: the exudate trap bottle was placed on a scale to measure the exudate suctioned, and a negative pressure transducer (PT) was connected to tube A to measure actual pressure at the wound.

The performance of the described low-cost negative pressure device was tested on a wound phantom that was built following a model described elsewhere [19]. A truncated cone-shaped wound (4.5 cm diameter, 2 cm depth), centered on a flat surface simulating healthy skin, was 3D printed (Figure 2A). The conic chamber included small holes to distribute a viscous fluid realistically mimicking wound exudate (Figure 2B), which was injected by a perfusion pump (PHD Ultra, Harvard Apparatus, Holliston, MA, USA) at a flow rate of up to 1000 mL/h, corresponding to a highly exuding wound of extended surface [20,21]. The exudate fluid was a solution of xanthan gum 0.1% in distilled water, having a realistic viscosity of 0.23 Pa·s [20]. An infrared light was placed on the wound phantom to maintain a physiologic temperature [20].

As shown in Figure 2C, the negative pressure device was connected to a common 0.5-L glass bottle set in a similar way to a liquid trap for the exudate and to the wound phantom dressing by means of conventional silicone or medical grade plastic tubes (≈4 mm ID). The device was set to different combinations and timings of intermittent negative pressure, and its performance was assessed by measuring two variables: pressure and exudate flow (Figure 2C). The actual vacuum pressure applied to the wound was measured by an external pressure transducer (26PC, Honeywell, Charlotte, NC, USA), and the exudate volume drained by the device was measured by a scale weighing the liquid trap bottle (density of simulated exudate: 1.03 g/mL (20)).

## 3. Results

**Figure 3:**
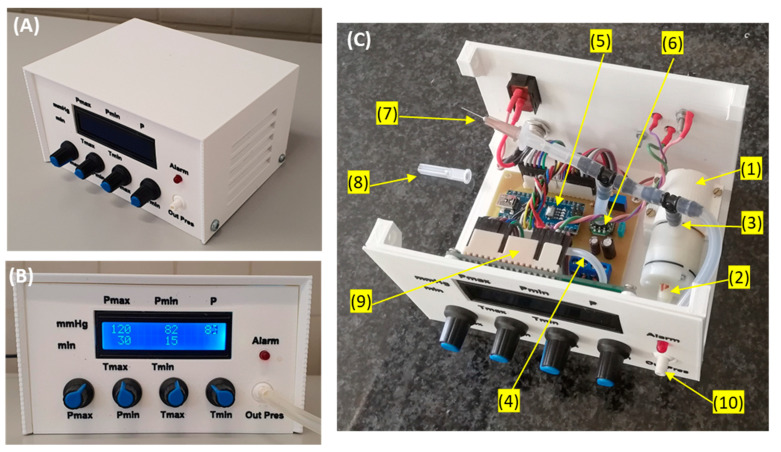
General (**A**) and front panel (**B**) pictures of the low-cost negative pressure device. Using knobs, the user can set the values and durations of maximum and minimum intermittent negative pressure (if these two values coincide or the time for one of the negative pressures is set to zero, constant pressure is generated). A display continuously shows the settings and the value of actual pressure (P) measured by the pressure transducer. A red-light alarm is on if vacuum pressure is outside a tolerance range. (**C**) Picture of the interior part of the device: (1) Negative pressure pump; (2): pump outlet; (3) pump inlet; (4) tube connecting room air with the pump outlet; (5) Arduino controller; (6): pressure transducer; (7): orifice (needle) open to room air; (8): perforated needle protector (for clarity, not in its place in this picture); (9) display; (10) negative pressure port of the device. See text for details.

Figure 3 shows a general (A) and a front panel (B) picture of the device. Its dimensions are 15 × 12 × 8 cm^3^ and its weight 460 g. The user can set the target vacuum pressure as continuous or intermittent by selecting two pressure levels (Pmax: range 25–175 mmHg; Pmin: range 0–Pmax), independently of each pressure value duration selectable within a range of 0–60 min. A red-light alarm for under/over pressure is triggered if the real-time measured pressure is outside the range 15–200 mmHg. Figure 3C is a picture of the interior of the device showing the implementation of the components in the diagram of Figure 1. To reduce the noise generated by the pump function, a silicone tube (12 cm length, 1 mm internal diameter, 3 mm external diameter) open to the air is connected to the pump outlet. Moreover, an additional mechanical safety system is set to ensure that in the case of a pump blockage, no permanent vacuum pressure is applied to the wound. To this end, a conventional 26 G ½” (0.45 × 13 mm) syringe needle at the pump inlet is placed open to the air room (Figure 3C).

The retail cost of the device material was ≤75 € (≈75 US$): 62 € for the components, including the pump, pressure sensor, microcontroller, digital display, power source, and all other electronic components, plus 10 € if including the material for enclosure 3D printing. Of note, the cost could be considerably reduced by wholesale purchasing.

**Figure 4:**
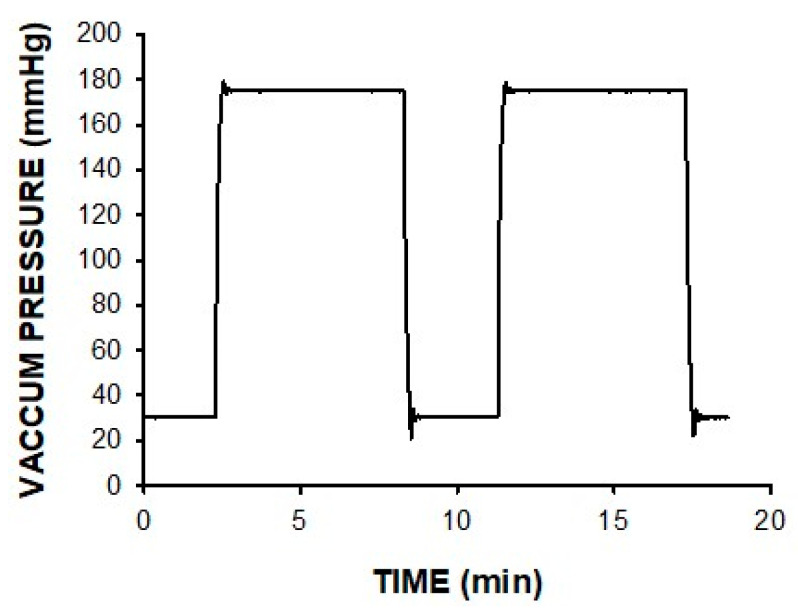
Vacuum pressure actually recorded at the wound phantom when the prototype of negative pressure device (Figure 1 and Figure 3) was set to an intermittent pattern consisting of 6 min at 175 mmHg and 3 min at 30 mmHg.

The device was able to generate the continuous or intermittent pressure pattern selected by the user. Figure 4 shows an example of the vacuum pressure actually applied to the wound for an intermittent pattern involving high/low pressures. Moreover, weighing the exudate flow showed that the device was able to perfectly drain the wound exudate for extremely high rates of up to 1 L/h.

## 4. Discussion

This report describes the design and bench-testing validation of a low-cost device to apply negative pressure for wound therapy in LMICs. It is a stand-alone device (not requiring an external negative pressure source) and enables us to set up any specific pattern of continuous or intermittent vacuum pressures. All the technical information necessary for assembling (and eventually modifying) the device is provided in full detail to be freely and unrestrictedly employed by any potential user. It is noteworthy that the device can be used regardless of the type of wound dressing materials employed, either conventional commercial dressing packs for negative pressure therapy or alternative simplified dressings adapted to the specific user availability [2,16,17,18]. It should be mentioned that the device described here outperforms previous low-cost proposals that only allow for the implementation of continuous vacuum pressure application [2,16,17,18]. Considering that the present technical proposal facilitates access to health becoming within reach of anyone, it fulfills the criteria stipulated in the definition of frugal innovation [22,23]. However, and contrary to most frugal innovations, the device does not exhibit reduced performance since it offers the same selectable options and quality performance as those available in commercial medical devices.

The negative pressure device described here is based on a modular structure (Figure 3C) requiring only basic electronic training for an overall straightforward and easy assembly. The design enables the simple replacement of each independent module since they are interconnected by compact electrical wiring. Significantly, assembling and using the device require no need for calibration routines since the pressure transducer is thermally corrected by construction and is provided with factory calibration. Interestingly, an automatic routine set in the microcontroller digitally corrects any potential drift in the zero signal of the pressure transducer each time the device is switched on. It should also be mentioned that the device has an electronic alarm system that triggers a red light when the pressure measured by the transducer is higher than a safety threshold of 200 mmHg or lower than 15 mmHg indicating pressure loss. On the other hand, an orifice (hypodermic needle) open to the air room and placed close to the vacuum pump outlet ensures that in the event of pump blockage, the wound will not remain under permanent negative pressure.

Nowadays, when it may seem that most therapies can only be applied by using expensive devices provided by a very competitive and specialized industry, the simplicity and performance of this low-cost device remind us of the need to focus on basic and simple designs that are easy to implement even in low-resource regions [24,25,26,27,28,29,30]. Interestingly, the approach proposed here for the construction of the negative pressure device for wound healing empowers the final users in LMICs to (a) fully control its construction procedure (e.g., modifying ranges of setting and alarms), (b) allow device servicing and repair at local reach and cost, (c) adapt the design based on local constraints (for instance replacing the 3D-printed enclosure with a conventional box, or changing the power supply from a 120/220V-AC-12V-DC transformer to a conventional 12 V car battery), and (d) to update the used components in response to market availability. A detailed explanation and discussion of design details can be found in the Supplementary material.

It is worth noting that the technical complexity required to assemble the device is at the level of a simple academic exercise for engineering/electronics students. In this regard, a possible option for clinicians potentially interested in using the device on their patients is to contact teaching staff in the technical schools in their region and foster collaboration. For such engineering professionals, helping to construct the device would be a straightforward and simple set of steps that could, for instance, be part of an example of practical work to be realized by students. Such collaboration may be synergistic for both sides since electronics/engineering partners would produce useful devices applicable in the real world, and the clinicians would have access to therapeutic tools that otherwise would be unavailable. Regardless of the construction approach, as the device can be assembled locally, the only costs incurred would be low since they involve purchasing general-purpose components (e.g., by e-commerce) and the relatively low labor costs in LMICs. Of note, the example proposed here may not only allow for adequate availability of negative pressure devices to patients [31,32,33] but may also help develop the local industry network in LMICs [34,35,36].

One of the main innovations and inherent advantages of the device described in the present study is that it is low-cost, easy to build, and more importantly, is provided with all technical details for assembly without any restrictions or intellectual property limitations (Supplementary materials). As such, we make it easy and possible to take advantage of the free open-source approach for locally reproducing and servicing the device by any potential user, particularly in LMICs [37]. As shown by the bench testing experiments, the device performs similarly to typical, commercially available devices with a cost that is ≈2 orders of magnitude cheaper [38]. As its design is open access, the device can be easily adapted to specific requirements that are more suitable to local environments. For instance, in cases where a 3D printer is not available, any conventional box can be used as a device enclosure. Moreover, in cases where the device is not designed for hospital use (as it is now) but for home treatment, all of the external setting buttons and displays can be eliminated by minimal microcontroller code changes (further reducing costs). However, a limitation of this report is that, besides its excellent performance on the bench, the device has not been tested in clinical applications as part of a highly supervised and costly clinical trial, which is a necessary next step for further implementation within countries aiming to adopt the technology for use in patient settings.

## 5. Conclusions

In conclusion, we have designed a low-cost negative pressure device with broad function capabilities and excellent performance, facilitating the spread of this wound healing therapeutic modality to patients in low-resource regions.

## Data Availability

All detailed information and data are provided in Supplementary materials.

## Supplementary Materials

The following supporting information can be downloaded at: https://www.mdpi.com/article/10.3390/jcm11185417/s1, Technical_Description.zip.
