# Supplementary material for: Device for Negative Pressure Wound Therapy in Low-Resource Regions: Open-Source Description and Bench Test Evaluation
Source: J Clin Med. 2022 Sep 15;11(18):5417. doi: 10.3390/jcm11185417 (PMC9503864; doi:10.3390/jcm11185417)
Supplement: Supplementary file 1 [file jcm-11-05417-s001.zip › Supplementary_Material_Negative-pressure/Technical_Description-Supplementary.pdf]

***DEVICE FOR NEGATIVE PRESSURE WOUND THERAPY IN LOW-RESOURCE REGIONS: OPEN SOURCE DESCRIPTION AND BENCH TEST EVALUATION***

# TECHNICAL DESCRIPTION

## DISCLAIMER

**This document provides technical information on the specific device prototype described in the publication indicated above.**

**Using components different from (although similar to) the ones described here may require technical adjustments or adaptations and therefore the final performance of the resulting device must be specifically assessed.**

**The authors of this document are not responsible for the use of the information contained herein nor for any device built using such information.**

## LICENSE

**This device is released under CERN Open Hardware License (OHL) v1.2 and all software and documentation under GNU General Public License (GPL) v3.0.**

**Content:**

1. Device description
2. Schematics of the circuit
3. PCB board layout
4. List of components
5. Connections
6. Enclosure by 3D printer
7. Arduino code
8. Techincal comments

## 1. Device description

### Front view:

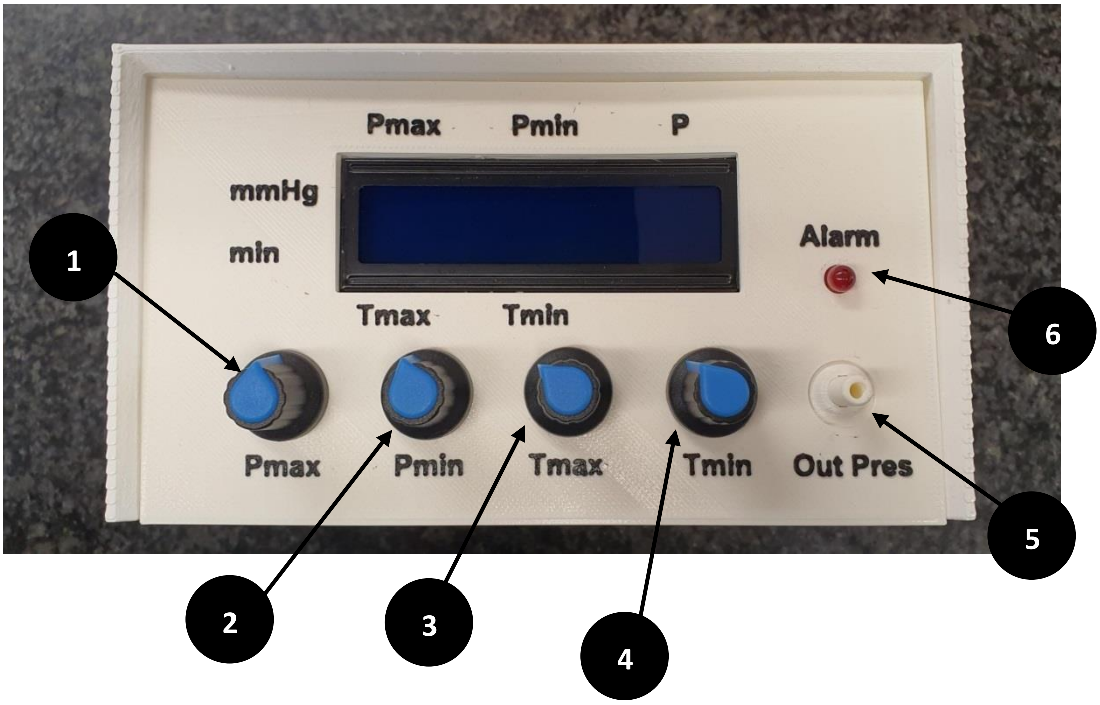


| Number | Device Feature | Description |
|---|---|---|
| 1 | Button to select the maximum pressure (Pmax) | Adjust Pmax (range 25 - 175 mmHg) |
| 2 | Button to select the minimum pressure (Pmin) | Adjust Pmin (range 25 mmHg - Pmax) |
| 3 | Button to select the time of Pmax (Tmax) | Adjust Tmax (0 - 60 min) |
| 4 | Button to select the time Pmin (Tmin) | Adjusts Tmin (0 - 60 min) |
| 5 | Vacuum Outlet Port | To connect the tubing to the exudate trap and wound dressing. |
| 6 | Alarm light (for over or under pressure) | Alarm on if actual pressure (P) is <15mmHg or >200mmHg |

**Rear view:**

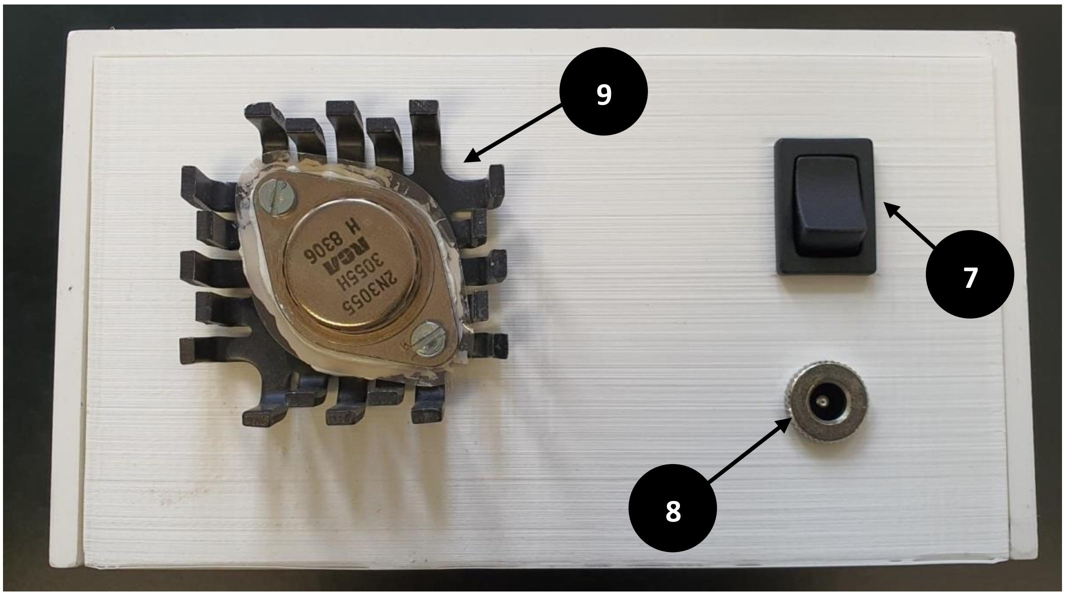


| Number | Device Feature | Description |
|---|---|---|
| 7 | ON/OFF | To set power on/off |
| 8 | Power inlet | To connect the external power source (12 V-DC) |
| 9 | External power transistor | Power dissipation |

## Conventional external power source (12 V DC; 1.5A)

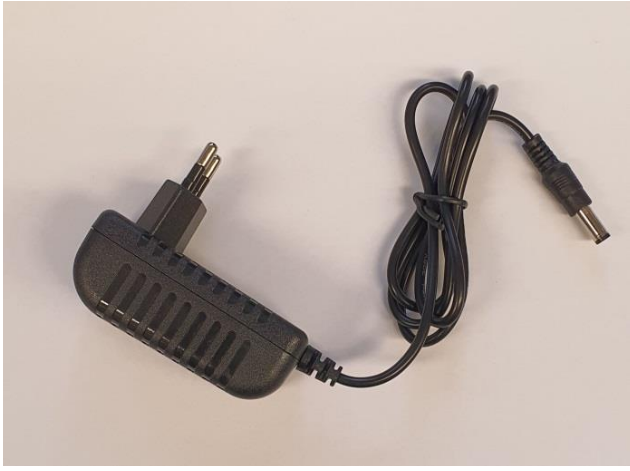

**Setting the negative pressure:**

- Connect the device using the external power source (8, rear).
- Switch the ventilator on (7, rear)
- The device will take a few seconds to auto-check and auto-correct any zero drift in the sensors. Note: Do not touch or use the device during this initial stage:

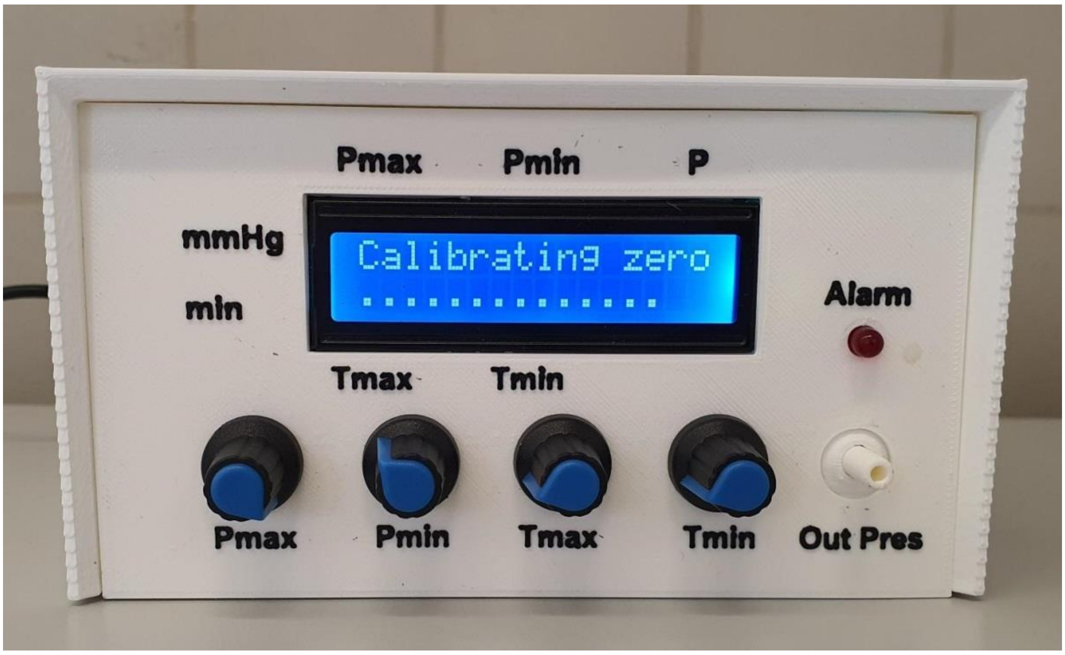


- When auto-check is finished, the following display appears. Pmin, Pmax, Tmin, Tmax can be set. Actual pressure (P) is shown in real-time:

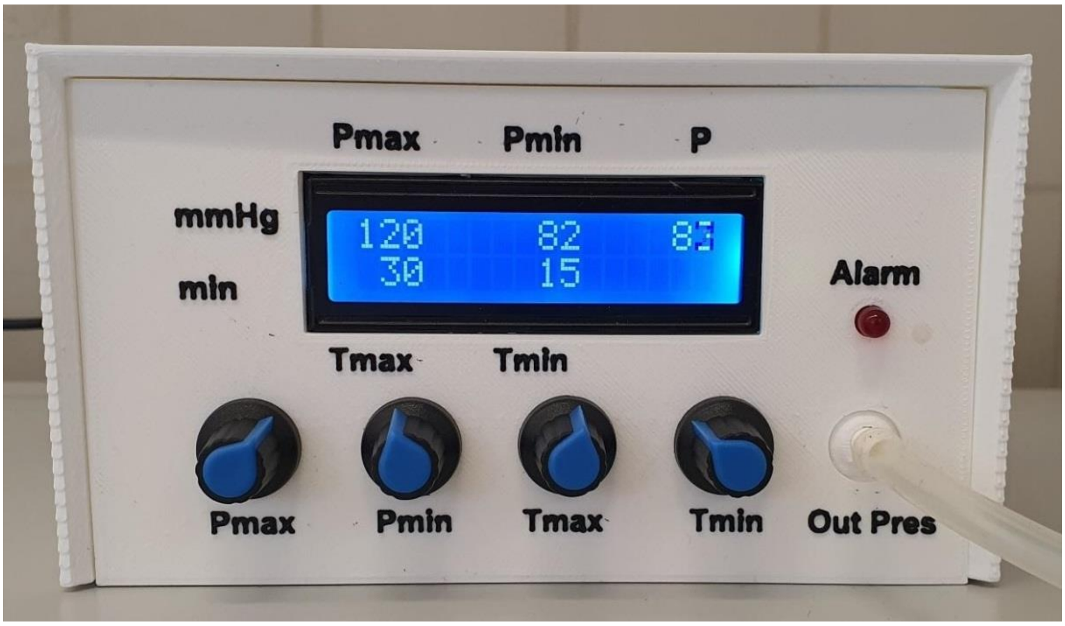

## 2. Schematics of the circuit

The source file **Negative-pressure_circuit.ddb** (included in the same zip folder where this pdf file is located) was written with Protel 99 SE and can be also read using https://www.altium.com/circuitmaker

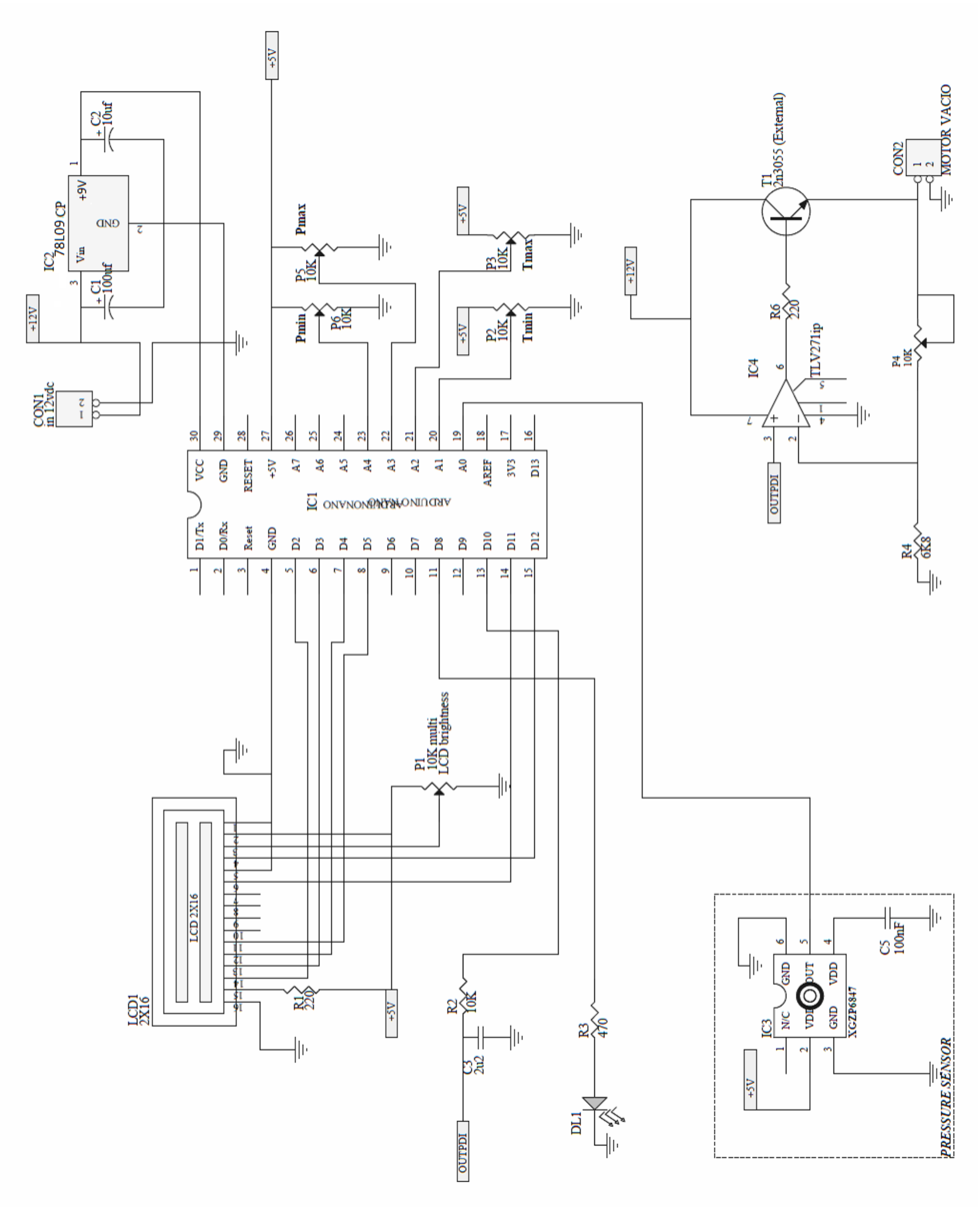

## 3. PCB board layout

The source file **Negative-pressure_circuit.ddb** (included in the same zip folder where this pdf file is located) was written with Protel 99 SE and can be also read using https://www.altium.com/circuitmaker

**Real SIZE: 60 X 80 mm**

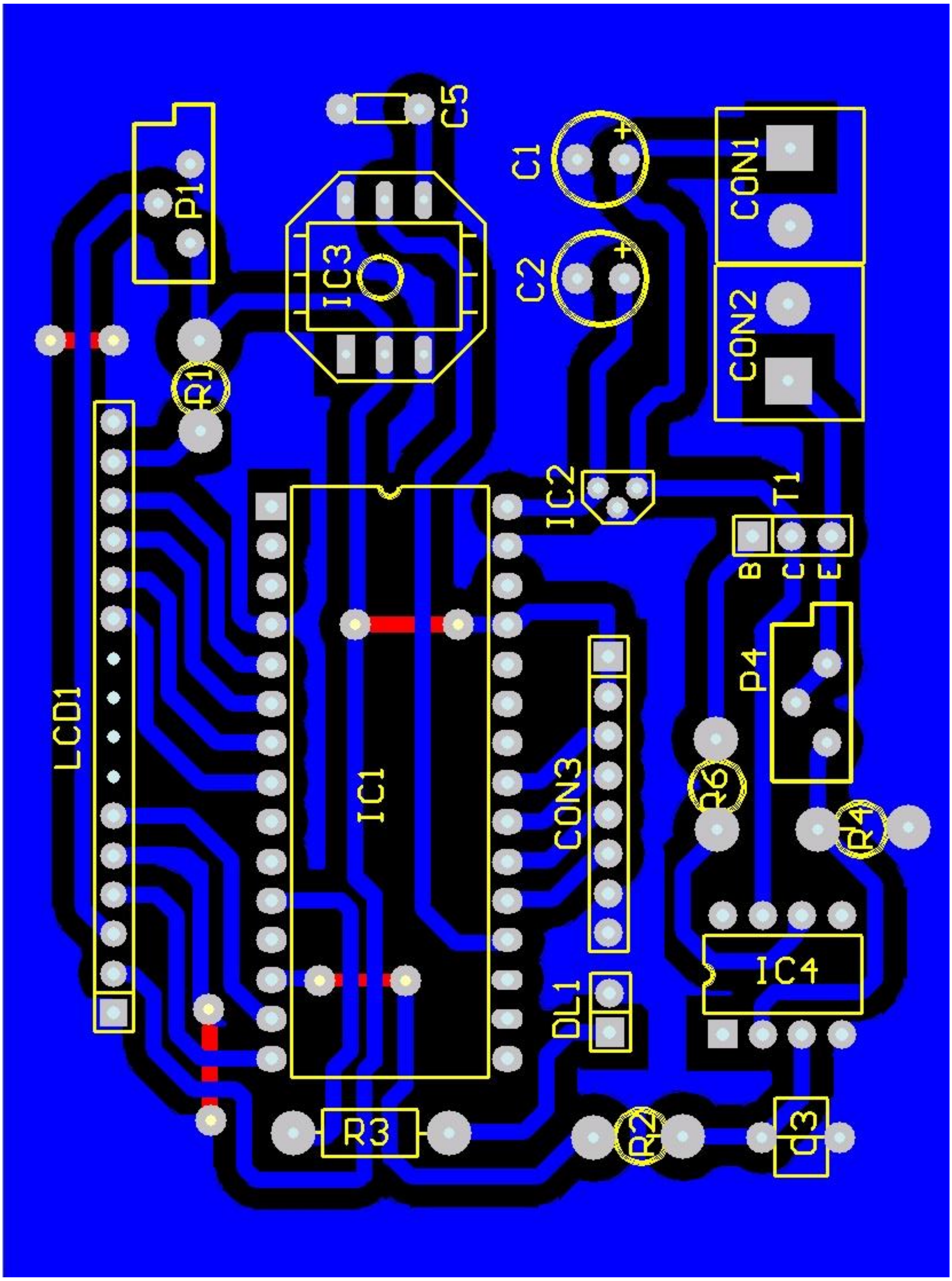

**Real SIZE: 60 X 80 mm**

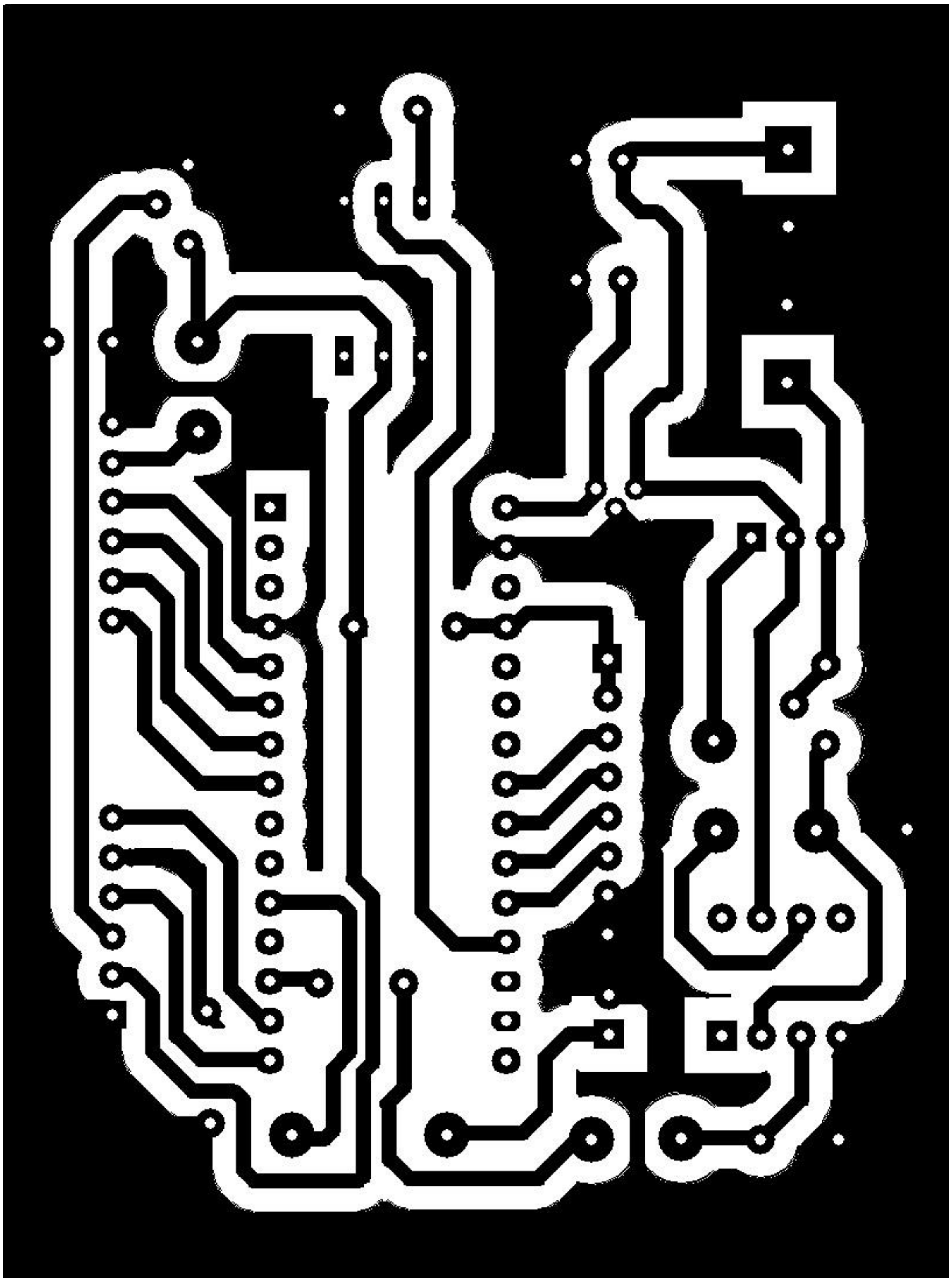

## 4. List of components

PCB:

- Resistors: 1x10kΩ, 1x470Ω, 1x6,8kΩ, 2x220Ω, 2x10kΩ potentiometer
- Capacitors 1x100µF (electrolitic), 1x10µF(electrolitic), 1x100nF
- 1x XGZP6847030KPGN pressure sensor (-30 ～ 0 Kpa)
- 1x MC78L09CP low-current voltage regulator
- 1x 2N3055 to3 power transistor
- 1x radiator to3
- 1x insulating mica to3
- 1x DIP32 Arduino Nano controller
- Connectors (2,54mm): 2x6pin, 1x8pin, 1x3pin, 1x2pin.
- Connectors (5mm): 2x2pin
- 1xTLV271IP operational amplifier (rail to rail)
- 1x DIP8 Socket
- Printed Circuit Board PRE-SENSITIZED POSITIVE (+) 1 SIDE (60mmx80mm)

External:

- 1x SODIAL(R) LCD display (model 015085) with HD44780 controller + 2x6pin connectors
- 4x HSEAMALL HB20 10kΩ potentiometers (16x7mm)
- 3 pin switch 1.3x0.8x3.3 cm
- 1x ON/OFF switch 22mm
- 1x LED 5mm head
- 1x1 Conventional external power source (12 V DC; 1.5A):

## 5. Connections

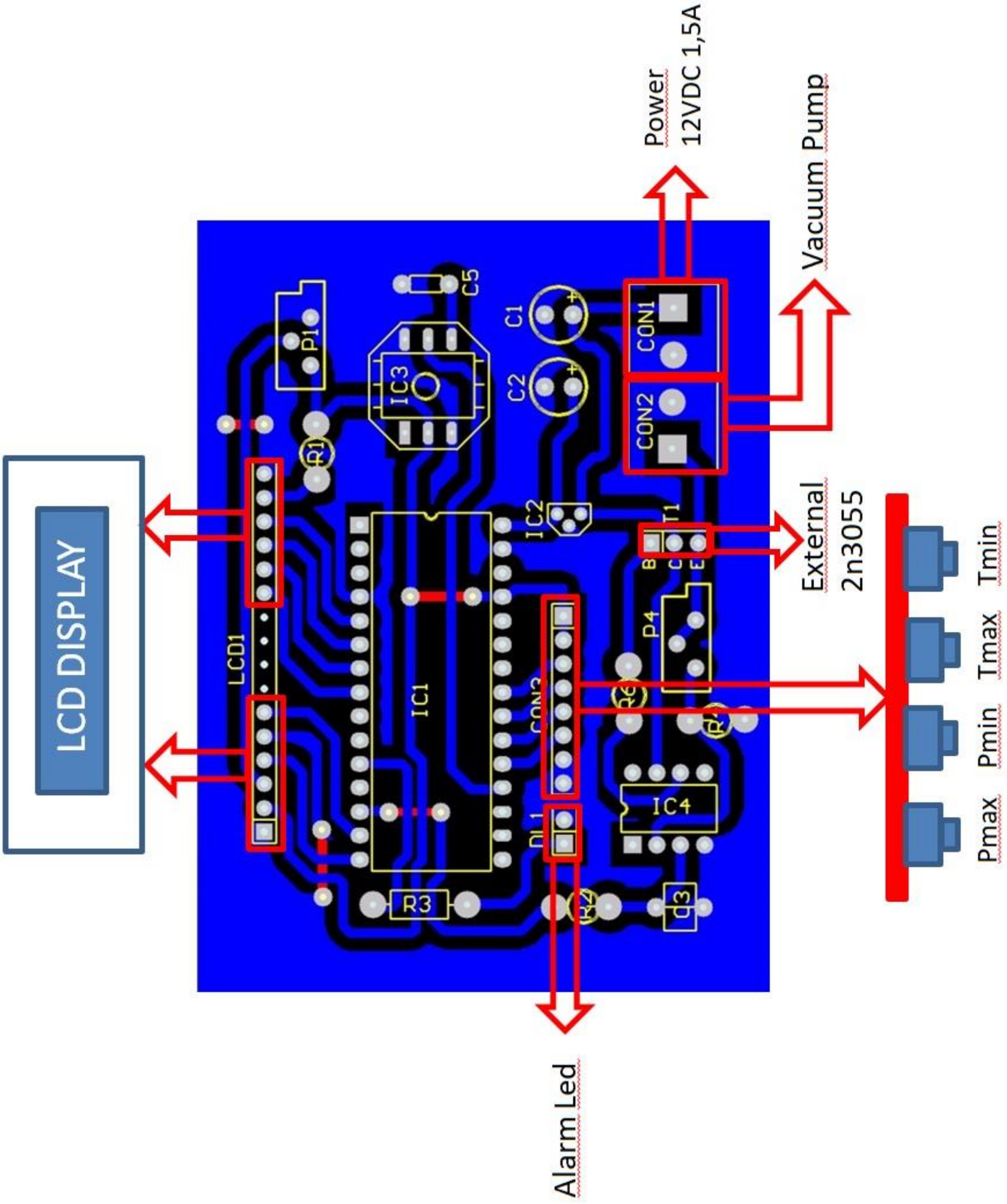

## 6. Enclosure by 3D printer

STL files for 3D printing in: **Negative-pressure_enclosure.zip** (included in the same zip folder where this pdf file is located).

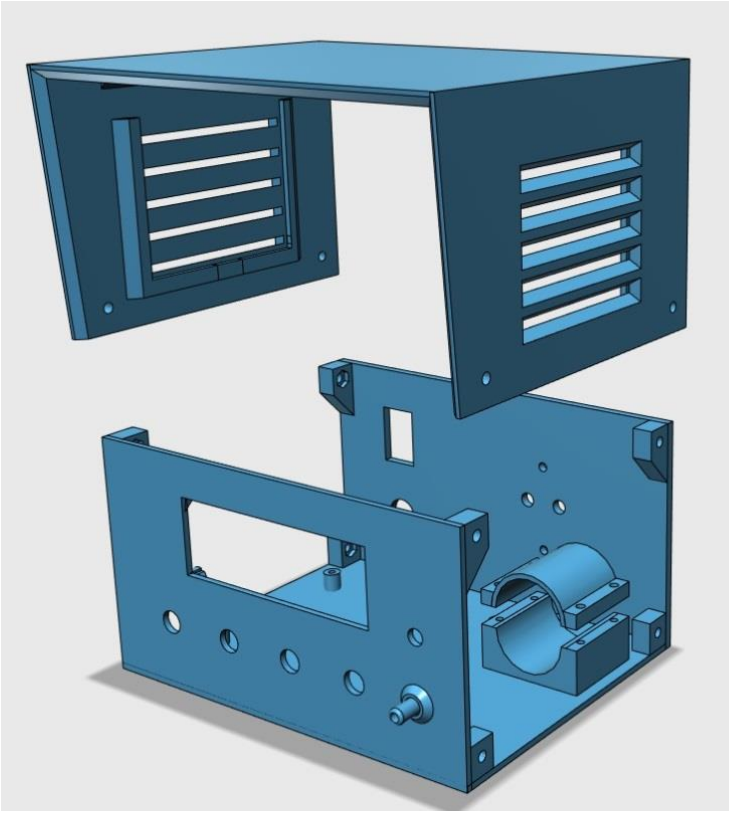

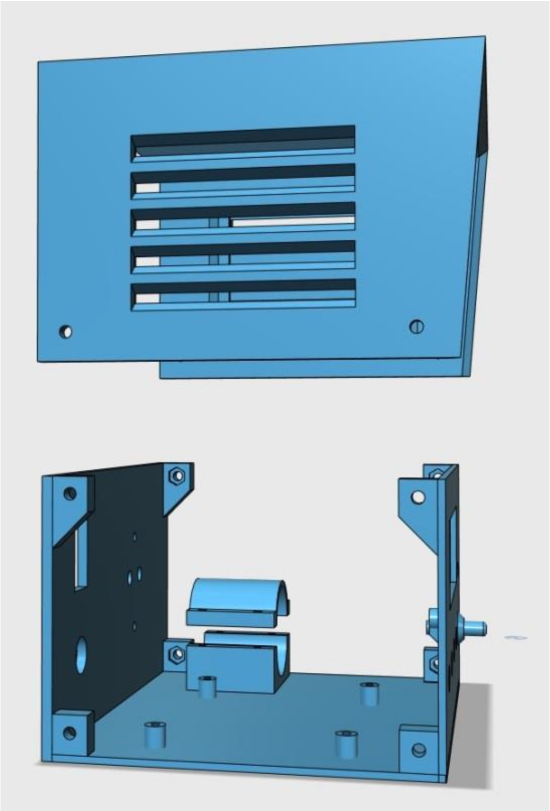

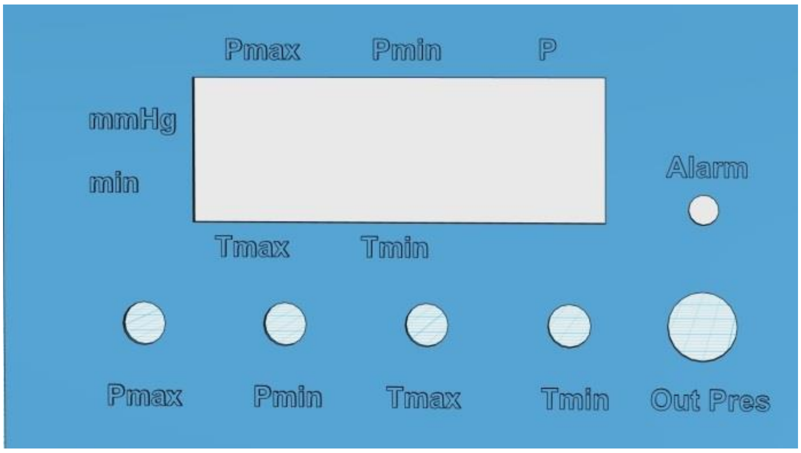

## 7. Arduino code

Source code in the file: **Negative-pressure_arduino.ino** (included in the same zip folder where this pdf file is located).

.

## 8. Technical comments

It should be mentioned that the design described herein is one among several options that could be chosen. We selected this specific solution based on simplicity and material availability.

1) The employed vacuum pump was chosen because it is off-the-shelf and easily available by e-commerce at a low cost. Its nominal maximum power supply is 12 V and it can generate vacuum up to 400-500 mmHg (airflow of 120-180 L/h), thus exceeding the requirement for the device. Therefore, we chose to power the pump at 5 V. This option has the advantage that the noise caused by the pump function is greatly reduced. To adjust this maximum voltage to 5V, the value of P4 must be set to 0 Ω. In this case, the system formed by IC4, P4, R4, and T1 has a gain equal to one, and hence, as the maximum output voltage of Arduino (OUTPDI) is 5V, this will be the voltage powering the vacuum pump (con2). The maximum gain that is obtained by adjusting the P4 potentiometer is approximately 2.4, so if it is adjusted to the maximum value (10 kΩ), the vacuum pump powering will be 5 V x 2.4 = 12 V.

2) To avoid including a valve open to room air to allow for decreasing vacuum pressure when required, we used an open-air orifice by means of a conventional hypodermic needle. The type of needle was chosen to offer a sufficiently high resistance so as to reduce the airflow through it and thus the flow demand to the pump. If the resistance of the needle is lower, it is possible that in case of unintended air leaks at the wound dressing the level of vacuum pressure achieved could be lower than required. In the case of using a needle with lower resistance, it could be necessary to power the pump at voltages greater than 5V (by adjusting potentiometer P4. This would increase the noise caused by the pump function.

3) The external power source was chosen since 12 V is a widespread option and also because it can be trivially replaced by a conventional car battery (in regions with compromised mains power supply). Alternatively, a power supply with a lower voltage (e.g. 9 V, 1.5 A) could be used. In this case, the 78L09 regulator (IC2) should be removed and terminals 1 and 3 corresponding to IC2 on the printed circuit board (PCB) should be short-circuited.

4) Potentiometer P1 determines the light intensity of the LCD screen. It should be adjusted as required.
